# Family Matters: Trauma and Quality of Life in Family Members of Individuals With Prader-Willi Syndrome

**DOI:** 10.3389/fpsyt.2022.897138

**Published:** 2022-06-28

**Authors:** Anja Bos-Roubos, Ellen Wingbermühle, Anneloes Biert, Laura de Graaff, Jos Egger

**Affiliations:** ^1^Centre of Excellence for Neuropsychiatry, Vincent van Gogh Institute for Psychiatry, Venray, Netherlands; ^2^Donders Institute for Brain, Cognition and Behaviour, Radboud University, Nijmegen, Netherlands; ^3^Department of Human Genetics, Radboud University Medical Centre, Nijmegen, Netherlands; ^4^Department of Internal Medicine, Erasmus University Medical Center, Rotterdam, Netherlands; ^5^Center for Adults With Rare Genetic Syndromes, Erasmus University Medical Center, Rotterdam, Netherlands; ^6^Dutch Center of Reference for Prader-Willi Syndrome, Rotterdam, Netherlands; ^7^Stevig, Specialized and Forensic Care for People With Intellectual Disabilities, Dichterbij, Oostrum, Netherlands

**Keywords:** trauma, PTSD, contextual neuropsychology, family, Prader-Willi syndrome (PWS), quality of life, systemic approach

## Abstract

**Background:**

Prader-Willi syndrome (PWS) is a potentially life threatening, genetic developmental disorder that requires lifelong medical treatment and behavioral management. PWS has a major impact on the patient's social environment. In this study, we have explored traumatic life events and symptoms of posttraumatic stress disorder (PTSD) in family members of individuals with PWS. We have also assessed quality of life in relation to trauma manifestations. In addition, we have evaluated demographic characteristics such as living setting of PWS patients as well as PWS symptom severity.

**Methods:**

Data of this observational study were obtained by means of the Life Events Checklist DMS-5, the Posttraumatic Stress Disorder Checklist DSM-5, the abbreviated World Health Organization Quality of Life questionnaire, the Lancashire Quality of Life Profile questionnaire, and a short demographic inventory. The study sample includes 98 adults aged 19 to 80 years (*M* = 49, *SD* = 15), who are relatives of 69 individuals with PWS aged 0 to 58 years (*M* = 19, *SD* = 13). Participants were recruited via the two Dutch patient associations PWS and the Dutch Digital Center of Expertise PWS.

**Results:**

Life time prevalence of traumatic events (93%) was higher in family members of PWS patients (“PWS relatives”) than in the general Dutch population (81%). Of those who reported any traumatic event, almost half reported *PWS-related* events. The prevalence of probable PTSD was higher in PWS relatives (12.1%) than the general lifetime prevalence of PTSD (worldwide, and in the Netherlands 7.4%). Predominant trauma symptoms in PWS relatives were “negative changes in arousal and reactivity” and “negative changes in cognition and mood;” both significantly negatively related to quality of life. Symptom severity of PWS individuals, as well as the associated trauma symptom severity of their relatives increased with age of the PWS individual. The presence of trauma symptoms was less frequent among relatives of PWS individuals living in a care facility.

**Conclusions:**

Having a relative with PWS is associated with higher prevalence of traumatic experiences and greater vulnerability to PTSD. Raising awareness in health care professionals of trauma symptoms in PWS relatives may contribute to effective treatment of their psychosocial stress. In addition, timely interventions might prevent family members from developing psychopathology like PTSD.

## Introduction

Prader-Willi syndrome (PWS; OMIM #176270) is a genetic neurodevelopmental disorder, caused by an anomaly in the paternally derived long arm of chromosome 15. PWS is characterized by multiple physical, cognitive, behavioral, and psychiatric symptoms. Hypotonia and pituitary hormone deficiencies, particularly hypogonadism occur most frequently (1–3). After birth, patients suffer from feeding difficulties, often necessitating tube feeding. Later in childhood, patients typically develop hyperphagia. In the absence of external control, this can result in excessive eating behaviors including pica, and consequent risks of developing obesity, diabetes mellitus, cardiopulmonary disease, and other serious somatic comorbidities (1, 4, 5). While PWS requires lifelong medical treatment and behavioral management, overall mortality rate is estimated at 3% per year and the average age at time of death is 40–50 years (6, 7).

As to cognitive and behavioral characteristics, PWS presents with mild to moderate intellectual disabilities, executive and social cognitive impairments and symptoms from the autism spectrum such as general cognitive inflexibility with perseverations, repetitive and ritualistic behaviors, temper tantrums, and self-injury (e.g., skin-picking). Challenging behaviors typically increase from teenage years up to their thirties, and affective disorders, mood instability or psychosis may frequently occur from adolescence onwards, albeit with varying severity (8–10).

PWS also has a major impact on PWS relatives. Lower levels of quality of life, increased burden of care and family problems have been reported in primary caregivers of young children with PWS (11–13). Furthermore, there is evidence of increased levels of distress and mood disturbances in family members (mostly mothers), as well as symptoms of posttraumatic stress disorder (PTSD) in siblings of young PWS patients (12, 14, 15).

So far, no information is available on trauma related distress and quality of life regarding wider family members related to PWS individuals in all chronological ages. This study aims to identify possible psychopathology and any underexposure of the issue in the group of family members. To this end, in those PWS relatives, we studied (a) traumatic life events and symptoms of PTSD, (b) trauma symptom severity and PWS-related stressors, (c) trauma symptom severity and Quality of Life, (d) age of PWS individuals and PWS symptom severity, the amount of PTSD symptoms, and Quality of Life respectively, and (e) living situation in relation to the occurrence of trauma symptoms, and content of reported trauma (see Table 1 for the specific hypotheses).

**Table 1:** Hypotheses.

**Research question**	**Hypothesis**
(a)	Family members will experience PWS related events as a traumatic stressor and will suffer from higher levels of trauma symptoms than the general population, to an extent that they will meet the criteria of PTSD (DSM-5; American Psychiatric Association, 17).
(b)	Trauma symptom severity will be positively related to the experience of PWS-related trauma.
(c)	Trauma symptoms severity will be inversely correlated with experienced quality of life. This third hypothesis will be further investigated by studying the relationship between distinct clusters of trauma symptoms and domains of quality of life.
(d)	Relatives of PWS individuals aged 10 to 30 years will report higher degrees of PWS symptoms, more experienced PTSD symptoms, and lower quality of life than relatives of PWS individuals aged 0 to 9 years and PWS individuals aged older than 30 years.
(e)	Relationship between living situations and the occurrence (presence or absence) of trauma symptoms, and content of reported trauma (PWS-related or not) will be explored.

## Materials and Methods

### Participants and Procedure

The study sample included data of 98 adult family members aged between 19 and 80 years (*M* = 49.5, *SD* = 15.0). Of them, 67 participants were first-degree relatives (20 fathers, 46 mothers, and 1 stepfather) and 31 participants were second-degree family members (6 brothers, 19 sisters, 1 grandfather, and 5 grandmothers) of a patient with PWS. These participants were the relatives of 69 PWS patients between the ages of 0 and 58 years (*M* = 20.4, *SD* = 13.6; 50% females). All participants were Dutch speaking. Table 2 presents additional information of both the family members and the PWS patients.

**Table 2:** Characteristics of family members and individuals with PWS.

**Variable**		
	***n***	**%**
**Family members**		
**Sex**		
Female	69	70.4
Male	29	29.6
**Nationality**		
The Netherlands	88	89.8
Belgium	9	9.2
Switzerland	1	1.0
**Living with the PWS patient**		
Yes	49	50.0
No	49	50.0
**Highest education completed with a diploma**^***a***^ **(corrected to Verhage code)**^***b***^		
No/special education (1)	0	-
Primary school (2)	1	1.0
Primary school and <2 years of low-level secondary school (3)	0	-
Low-level secondary school (4)	1	1.0
Average-level secondary school (5)	11	11.3
Average-level secondary school (5), High level secondary school (6)	21	21.7
High level secondary school (6)	44	45.3
University (7)	19	19.6
**Paid job**	66	67.3
**Experienced PWS symptom severity over the last 2 weeks** ^**c**^		
0	2	2.3
1	6	9.3
2	5	5.8
3	6	7.0
4	6	7.0
5	13	15.1
6	8	9.3
7	6	7.0
8	19	22.1
9	12	14.0
10	3	3.5
**Individuals with PWS**		
**Sex of the related PWS individual** ^**d**^		
Female	49	50.0
Male	49	50.0
**Living setting of the related PWS individual** ^**d**^		
Care facility	38	38.8
No care facility	60	61.2

^a^* n = 97. ^b^ Responses on question 1.4b of the Dutch version of the Lancashire Quality of Life Profile (16, 17). For the corrections for level of education, we used a seven-point scale ranging from 1 (primary school not completed) to 7 (academic degree) according to the Dutch educational system (18). This scale is comparable to the International Standard Classification of Education [UNESCO, (19)]. ^c^ n = 86. Scores on a eleven-point Likert scale (0 = no symptoms at all, 10 = symptoms to a very serious extent). ^d^ n = 69*.

Participants were recruited via the websites of the Dutch Digital Center of Expertise PWS (provisioning public information by the cooperating PWS patient associations and designated PWS patient care organizations), and of the two Dutch patient associations PWS (the Prader-Willi Fund, and the Prader-Willi Foundation). Further, invitation letters were deposited at the front desk for visitors of the Vincent van Gogh Centre of Excellence for Neuropsychiatry outpatient clinic, which provides shared care together with the Expertise Center for Adults with Rare Genetic Syndromes of the Erasmus University Medical Center. After the written provision of information about the research objectives and procedures, participants gave their written informed consent for voluntary and individual participation in the study. The study was performed in accordance with the Declaration of Helsinki and was approved by the Vincent van Gogh Institutional Review Board (Decision letter references: JE/hr/2020.012/; JE/hr/2021.004).

Questionnaires (provided with a reply envelope) were sent to the home addresses of the participants. Inclusion criteria were first degree (biological-, step-, and adoption parents) and second degree (biological siblings, step siblings, and grandparents) relationship with a PWS individual. Age criterion for those relatives was at least 18 years old, to legally ensure voluntary self-selection. Data about the related PWS individuals were obtained anonymously. As a convenience sample, data were collected in the period from September 2020 to July 2021.

### Materials

Questionnaires were provisioned on hard copy forms and self-completed. The total completion time was (based on a prior try out) estimated at 60 to 75 min.

#### Demographic and PWS Information

Demographic data were collected partly by the Dutch version of the Lancashire Quality of Life Profile (LQoLP) (16, 17), and additionally by a ten-items questionnaire designed for this study. This form records participants' family relationship to and information of the PWS patient, such as chronological age and living setting. The latter evaluates the experienced severity of the PWS symptoms by relatives in the previous 2 weeks on a eleven-point Likert scale from 0 (*no symptoms at all*) to 10 (*symptoms to a very serious extent)*.

#### Trauma

To measure trauma symptoms according to the Diagnostic and Statistical Manual of Mental Disorders (DSM-5; (20)) the Dutch versions of the Posttraumatic Stress Disorder Checklist for *DSM-5* (PCL-5-NL, (21, 22)) and the Life Events Checklist for *DSM-*5 with extended A-criterion (LEC-5-NL, (21, 22)) were used. The first part of the LEC-5-NL screens for the occurrence of traumatic events in a participants' entire life. It consists of 17 items; assessing exposure to 16 major and/or stressful PTSD relevant events (e.g., natural disaster, sexual assault, life-threatening illness or injury) and one open item for reporting any other extraordinarily stressful event or experience that is not in the list. The second part comprises 9 items and checks the A criterion of PTSD (exposure to actual or threatened death, serious injury or sexual violence). If participants check for anything on the open item of part 1, first identification of that event is asked for (question A) in part 2. Subsequently, if more than one of the events are reported in part 1, a brief description of the currently worst event is asked for (question B) followed by the 7 remaining questions regarding that event of part 2. The third part is covered by the PCL-5-NL (see Supplementary Material). The PCL-5 assesses the number and severity of PTSD symptoms in the past month, while keeping the worst event in mind, i.e., intrusion symptoms (cluster B; items 1–5), avoidance symptoms (cluster C; items 6 and 7), negative alterations in cognitions and mood (cluster D; items 8–14), and negative alterations in arousal and reactivity (cluster E; items 15-20). Answers on these 20 items have to be filled out on a five-point Likert scale from 0 (*not at all*) to 4 (*extremely*); reflecting symptom occurrence and severity. The score per cluster varies between 0 and 28 (cluster B: 0–20; cluster C: 0–8; cluster D: 0–28; cluster E: 0–24). Mean PTSD symptom cluster scores were calculated as subtotals of the corresponding items. Items can be summed to provide a measure for overall symptom severity (range 0 to 80). A cut-off score between 31 and 33 is considered to be indicative of probable PTSD across samples, and suggests that the patient may benefit from PTSD treatment. A cut-off point of 33 represents a good predictor of a PTSD diagnosis (23). The reliability and validity of the PCL-5 are considered to be strong (24). The LEC-5 has adequate psychometric properties as well (25).

#### Quality of Life

Quality of life was assessed using the Dutch abbreviated version of the World Health Organization Quality of Life Questionnaire (WHOQOL-BREF-NL) (26, 27). This is a self-report questionnaire containing 26 items. The first two items evaluate the subjective overall quality of life and general health, respectively. From the other 24 items seven cover the quality of life domain physical health, six psychological functioning, three social relationships, and eight environment. Answers have to be given on a five-point Likert scale from 1 (*very poor*) to 5 (*extremely*). A higher score corresponds to a better quality of life. The quality of life domain scores were calculated as means of the underlying items. WHOQOL-BREF reliability has been rated as “good” to “excellent” and validity as “good” (28).

To also gain insight into particular objective aspects of quality of life, also, the extensive Dutch version of the Lancashire Quality of Life Profile was used [LQoLP; (16, 17)]. The LQoLP consists of 126 items and distinguishes the following nine different domains of quality of life: Work and education, Leisure and participation, Religion, Finances, Living situation, Legal status and safety, Family relations, Social relations, and Health. Each domain comprises both subjective and objective questions. In this paper, only the objective items are used, which must be answered categorically (*yes/no*). Internal consistency, test-retest reliability and validity of the LQoLP are considered to be good (17).

### Statistical Analyses

IBM SPSS version 27 for Windows was used for all statistical analyses. Due to missing responses, the number of participants differs across the various analyses.

First, life time prevalence of traumatic events was calculated based on Part 1 of the LEC-5-NL. To this end, the number of participants who had ticked one or more of the 17 options for experienced traumatic events were summated. Subsequently, the proportion of participants who reported PWS-related issues (e.g., traumatic birth, aggressive behavior, psychosis) as a traumatic life event on the forms was calculated (question A of Part 2 on the LEC-5-NL). Also, the proportion of subjects who considered the PWS-related event as currently the worst was established (question B of Part 2 on the LEC-5-NL). In addition, the proportion of subjects with a Total PTSD symptom on the PCL-5-NL at the cut-off point (33 and higher) was calculated.

Secondly, two groups were formed based on the content of the reported traumatic experiences on the LEC-5-NL: PWS-related trauma vs. other trauma. Subsequently, a two-tailed *t*-test for independent variables was performed with nature of trauma as (categorical) independent variable, and Total PTSD symptom score and the four PTSD symptom-cluster scores on the PCL-5-NL as dependent variables.

Thirdly, a Pearson's correlation coefficient was computed to assess the relationship between the Total PCL-5-NL score of trauma symptoms and the Total WHOQOL-BREF-NL score of the degree of quality of life in family members of patients with PWS. Additionally, the relationship between the four PTSD symptom clusters (B to E) on the PCL-5-NL and the four quality of life domains (physical health, psychological functioning, social relationships, and environment) on the WHOQOL-BREF-NL was analyzed. However, the PTSD symptom cluster scores are not assumed to be independent and are considered as non-categorial variables (range variables expressed as mean scores within the set of the real variable). Therefore, a GLM multivariate analysis of covariance (MANCOVA, Wilk's Lambda) test was performed with the distinct four mean PTSD symptom cluster scores as four covariates, and the distinct four mean quality of life domain scores as the linear combination of the dependent variables. A Bonferroni correction for multiple testing was applied to reduce the risk of a Type-1 error (29).

Fourthly, to investigate whether the perceived severity of PWS symptoms, experienced PTSD symptoms, and quality of life in family members differ between chronological ages of the PWS patient, subjects were divided into three age groups: family members of PWS individuals aged “0–9 years,” “10–30 years,” and “31 years and older.” A one-way analyses of variance (ANOVA) with a *post-hoc* Bonferroni correction was performed, with perceived PWS symptom severity (item 10 on the short form), experienced PTSD symptoms (total score and four mean scores per trauma symptom cluster on the PCL-5-NL), and experienced quality of life (total score and four means scores per domain on the WHOQOL-BREF-NL) as dependent variables, and age group as independent variable.

Finally, a two level categorical variable was formed based on the occurrence of trauma symptoms on the PCL-5-NL: family members who did not score any trauma symptom (*absence*) an who scored one or more trauma symptoms (*presence*). Likewise, a variable was formed based on the reported content of trauma on part B of the LEC-5-NL (*PWS-related trauma, and other trauma*). Additionally, two categorical variables regarding the residential setting were selected (“Do you live together with the PWS patient?;” “Does your relative with PWS live in a care facility?” on the Demographic inventory; *no/yes*). Four chi-squared tests for independence were applied to analyze successively if occurrence of trauma symptoms and content of trauma were associated with the residential setting of the PWS patients, and of the family members. *P*-values were calculated with Fisher's Exact Test.

## Results

Lifetime prevalence of traumatic events in family members of PWS patients was 92.9% (*n* = 91 out of 98). Seven participants (7.1%) reported no occurrence of any traumatic experience. Of those who reported traumatic events, 46.2% (*n* = 42) reported a PWS related experience as a traumatic event and 53.8% (*n* = 49) reported a traumatic experience of a different nature. Of the participants who reported a PWS-related traumatic event, 73.8% (*n* = 31) considered the PWS related event as currently the worst (currently bothering them the most). Furthermore, 11 of the 91 participants (12.1%) who reported traumatic events scored at or above the cut-off point of 33 on the PCL-5-NL, implicating that the severity of their current trauma symptoms probably indicates PTSD.

Participants who reported a PWS-related trauma scored significantly higher on both the total symptoms of PTSD of the PCL-5-NL, as well as on the four distinct clusters of PTSD symptoms of the PCL-5-NL compared to participants who experienced different, non PWS-related traumatic events. Results are displayed in Table 3.

**Table 3:** T-test of independent variables on PTSD symptoms scores in PWS-related trauma vs. other trauma.

**PTSD symptoms scores**	**PWS-related trauma**		**Other trauma**		***t*_**(91)**_**	***p-*value**	**Cohen's *d***
	**M**	**SD**	**M**	**SD**			
Total	18.76	13.64	10.61	12.13	2.988	0.004**	12.853
Intrusion	0.90	0.90	0.49	0.71	2.404	0.019*	0.805
Avoidance	1.02	1.15	0.50	0.89	2.403	0.019*	1.017
Negative alterations in cognition and mood	0.88	0.74	0.52	0.71	2.379	0.020*	0.722
Negative alterations in arousal and reactivity	1.00	0.73	0.59	0.60	2.922	0.005**	0.666

n = 91 (number of participants who experienced trauma during lifetime). Intrusion, Avoidance, Negative alterations in cognitions and mood, and Negative alterations in arousal and reactivity represent respectively the four distinct PTSD symptoms clusters B to E on the PCL-5-NL for DSM-5 (20). Mean PTSD symptom values are shown for the group who reported PWS-related trauma (n = 42) and the group who reported Other trauma (n = 49), as well as the results of the t test (assuming unequal variance) comparing the PTSD symptom scores between the two groups. ^*^p < 0.05. ^**^p < 0.01.

There was a significant and substantial negative correlation between total PCL-5-NL score of trauma symptoms and the total WHOQOL-BREF-NL score of quality of life (*r*_(94)_ = −0.57, *p*<*0.0*01, *R*^2^^=^ 0.33). There were significant differences in PTSD symptom clusters “cognition and mood” (*F*_(4, 86)_ = 5.589, *p* < 0.001, ${\eta_{\text{p}}}^{2}$ = 0.206), and “hyperarousal and reactivity” (*F*_(4, 86)_ = 4.124, *p* = 0.004, ${\eta_{\text{p}}}^{2}$ = 0.161). These two symptom clusters were further examined. Negative alterations in cognition and mood appeared to be inversely related with all four domains of quality of life (physical health, psychological, social relationships, and environment). Further, negative alterations in arousal and reactivity were negatively related to the quality of life domain physical health. These significant results are presented in Table 4.

**Table 4:** Regression coefficients of associations between quality of life domains (WHOQOL-BREF-NL) and PTSD symptom clusters (PCL-5-NL) by MANCOVA statistics.

**Variable**	***B***	***SE***	***t***	***p-*value**	**95% CI**	${\eta_{\text{p}}}^{2}$
**Negative alterations in cognitions and mood**						
**Quality of life domain**						
Physical	−1.45	0.59	−2.46	0.016*	[−2.6,−0.3 ]	0.06
Psychological	−1.53	0.40	−3.81	0.000***	[−2.3,−0.7 ]	0.14
Social	−1.59	0.62	−2.57	0.012*	[−2.8,−0.4 ]	0.07
Environmental	−1.47	0.37	−3.99	0.000***	[−2.2,−0.7 ]	0.15
**Negative alterations in arousal and reactivity**						
**Quality of life domain**						
Physical	−1.79	0.59	−3.05	0.003**	[−3.0,−0.6 ]	0.10
Psychological	−0.48	0.40	−1.20	0.235	[−1.2, 0.3 ]	0.02
Social	−0.37	0.61	−0.60	0.552	[−1.6, 0.9 ]	0.00
Environmental	0.44	0.37	1.19	0.236	[−2.9, 1.2 ]	0.02

n = 85. CI, confidence interval. Negative alterations in cognitions and mood, and Negative alterations in arousal and reactivity represent the PTSD Symptom Clusters D and E in the DSM-5 respectively (20). Cluster D symptoms comprise among others “having strong negative beliefs about yourself, other people, or the world;” “having strong negative feelings such as fear, horror, anger, guilt, or shame;” “loss of interest in activities that you used to enjoy;” “feeling distant or cut off from other people;” and “trouble experiencing positive feelings (for example, being unable to feel happiness or have loving feelings for people close to you).” Cluster E symptoms are for example “irritable behavior, angry outbursts, or acting aggressively,” “being ‘superalert' or watchful or on guard”, “feeling jumpy or easily startled,” “having difficulty concentrating,” and “trouble falling or staying asleep.”^*^p < 0.05. ^**^p < 0.01. ^***^p < 0.001.

Family members of PWS individuals reported more severe PWS symptoms in the PWS age groups “10–30 years” and “≥ 31 years,” compared to PWS individuals aged 0–9 years. PTSD symptom scores among family members of patients aged 10–30 years were significantly higher than in the youngest age group, for the Total PTSD-score of the PCL-5-NL, as well as on its four trauma clusters (intrusion symptoms, avoidance symptoms, negative alterations in cognition and mood, and negative alterations in arousal and reactivity). The Total PTSD score and the level of arousal and reactivity symptoms were significantly lower in PWS individuals over 30 years than in those aged 10–30 years. The results are presented in Table 5. Quality of life did not differ between age groups.

**Table 5:** Means, standard deviations, and one-way analyses of variance (ANOVA) between age of PWS patients on experienced PWS symptoms, and PTSD symptoms, in family members.

**Measure**	**Age**				**ANOVA**				
		***M***	***SD***	***n***	**Effect**	**Mean difference**	***F* ratio**	***p-*value**	**η^2^**
Experienced PWS-symptom severity		5.85	2.72	86			6.112	0.003**	0.128
	Group 1	4.29	2.56	24	Group 2	−2.17		0.004**	
	Group 2	6.47	2.38	43	Group 3	0.04		1.000	
	Group 3	6.52	2.97	19	Group 1	2.13		0.025*	
Total PTSD-symptoms		13.81	13.33	96			6.542	0.002**	0.123
	Group 1	8.31	6.67	26	Group 2	−10.11		0.004**	
	Group 2	18.42	14.72	48	Group 3	8.14		0.042*	
	Group 3	10.27	13.33	22	Group 1	1.97		1.000	
Intrusion		0.64	0.82	96			4.903	0.009**	0.095
	Group 1	0.35	0.39	26	Group 2	−0.54		0.018*	
	Group 2	0.89	0.67	48	Group 3	0.46		0.081	
	Group 3	0.44	0.70	22	Group 1	0.08		1.00	
Avoidance		0.70	1.03	96			4.674	0.012*	0.091
	Group 1	0.35	0.68	26	Group 2	−0.66		0.022*	
	Group 2	1.01	1.22	48	Group 3	0.56		0.096	
	Group 3	0.46	0.71	22	Group 1	0.11		1.000	
Negative alterations in cognitions and mood		0.66	0.73	96			3.762	0.027*	0.075
	Group 1	0.40	0.42	26	Group 2	−0.46		0.031*	
	Group 2	0.85	0.79	48	Group 3	0.30		0.32	
	Group 3	0.55	0.80	22	Group 1	0.16		1.000	
Negative alterations in arousal and reactivity		0.76	0.69	96			6.030	0.003**	0.115
	Group 1	0.51	0.50	26	Group 2	−0.48		0.010**	
	Group 2	1.00	0.73	48	Group 3	0.44		0.031*	
	Group 3	0.55	0.65	22	Group 1	0.04		1.000	

Group 1 consists of family members of PWS patients aged 0 to 9 years. Group 2 consists of family members of PWS patients aged 10 to 30 years. Group 3 consists of family members of PWS patients aged 31 years and older. Intrusion, Avoidance, Negative alterations in cognitions and mood, and Negative alterations in arousal and reactivity represent the four respective PTSD symptom clusters B to E on the PCL-5-NL for DSM-5 (20). The mean difference is significant at the 0.05 level. ^*^p < 0.05. ^**^p < 0.01.

A significant interaction was found between the occurrence of trauma symptoms in family members with PWS patients living in a care facility or not (χ^2^ (1) = 6.02, *p* = 0.021). More often the absence of trauma symptoms (73%) than the presence (34%) was reported among relatives of PWS patients living in a care facility. Another significant interaction was found between the content of the experienced trauma (LEC-5-NL) and living setting of the family member (χ^2^ (1) = 4.99, *p* = 0.035). Family members who reported a PWS-related traumatic event (64%) more often lived with their relative with PWS than family members who reported other traumatic events (41%).

## Discussion

This first study on trauma and distress in adult relatives of individuals with PWS shows an increased risk for traumatic experiences and greater vulnerability to PTSD. As expected, we have found higher life time prevalence of traumatic events in adult family members than in the general Dutch population (92.9 vs. 80.7%) (30). Of those who reported any traumatic event, almost half reported *PWS-related* events. Furthermore, the prevalence of probable PTSD in this study (12.1%) exceeded the general lifetime prevalence of PTSD (worldwide between 1.3 and 8.8%; (31)) and in the Netherlands (7.4%; (30)). These findings are in line with previous research which showed that a severe or chronic illness of a relative can be experienced as a traumatic stressor and has been associated with manifestations of PTSD symptomatology (32–34). In addition, as hypothesized, family members who reported *PWS-related* trauma scored higher on PTSD symptoms than participants who experienced a different, non PWS-related traumatic event. These findings should increase clinical awareness of signs of psychopathology, and result in a recommendation to systematically monitor the experience of traumatic events in relatives of patients with PWS.

As expected, there was a negative relation between trauma symptoms and quality of life; particularly with respect to negative alterations in arousal and reactivity (cluster E), and in negative alterations in cognition and mood (cluster D). Cluster E symptoms were specifically negatively correlated to physical quality of life. The physical aspects of (acute and chronic) stress (high adrenaline and/or cortisol levels, sleep issues) could contribute to this relationship. Cluster D symptoms were negatively and fairly evenly related to all quality of life domains, affecting the experienced physical health, psychological health, social relationships, and living environment simultaneously. These results encourage awareness of clinicians regarding reciprocal influences of quality of life and the forementioned particular trauma symptoms in family members of PWS individuals. Cure of PTSD symptoms in family members might be supported by care in different life domains.

Further, results showed that higher chronological age of the PWS individual was related to increased PWS symptom severity evaluated by the family members. Both the total trauma score as well as the respective scores in all different trauma symptom clusters (B-E) were significantly higher in family members of the PWS patient age group 10 to 30 years (adolescents and young adults), compared to the age group 0 to 9 years (childhood). These findings regarding chronological age, PWS symptom severity and PTSD symptom severity did not fully demonstrate the expected quadratic relationship, since the expected PWS- and trauma severity differences between the age groups 2 and 3 did not reach significance. A possible explanation could be admittedly lower degree of challenging behaviors in PWS patients in group 3 (35), but higher levels of physical problems in this PWS patient age group (36), which may also cause stress and psychological complaints in caregivers (37). Nevertheless, as expected, increasing age of the PWS patient (from 10 years and up) was related to both PWS symptom severity and PTSD symptom severity negatively. This finding endorses the importance of clinical attention to both advanced care planning for the individual PWS patient, and timely support for their family members.

Finally, living together with their PWS relatives increased the risk of trauma symptoms, also of PWS-related trauma. Follow up research could explore trauma related symptoms in family members in relation to the intensity of their involvement in daily care of an individual with PWS, and the experienced caregiver burden.

Several limitations can be identified in the current study that may limit the generalizability of the results. At least the following two have to be mentioned here. Firstly, while selection-bias is difficult to avoid in research on genetic developmental disorders, due to participant recruitment by volunteer sampling, an over-representation of family members who felt attracted to the research topic may have occurred. Secondly, the sampling period was during the COVID-19 pandemic, which may have had a pre-existent negative impact on the experienced quality of life and health (38). Nevertheless, this applies to all participants who participated and leaves aside the differences we have found within our study population. Notwithstanding these limitations, the strengths of the study concern the considerable sample size, and the wide age range of related PWS individuals (from infancy to late adulthood), enabling cross sectional analyses. Furthermore, participation concerned all adult family members, instead of parents only. Demographic data regarding participants' educational levels indicate a fairly distributed representation, but a tendency to the higher end. At the same time, a majority of female relatives (~2/3) was involved in this study. Both aspects could both under- and over-estimate the results, since distress can be considered a product of a dynamic transaction between individuals (including cognitive, physiological, affective, psychological, neurological systems) and their complex environment; consistent with the transactional model of stress and coping (39).

The results presented in this study warrant further, preferably longitudinal, research. When compared to other rare genetic developmental disorders, such as Williams syndrome, Fragile X syndrome, or Smith-Magenis syndrome, parents of patients with PWS have shown higher levels of stress (40, 41). Future research could compare our results with those on trauma and quality of life in relatives of patients with other genetic developmental disorders.

## Conclusion

Having a relative with PWS is associated with higher prevalence of experienced traumatic events, and of PTSD, affecting the wider family system. Raising awareness in health care professionals of the typical presentation of trauma symptoms in PWS relatives may contribute to effective treatment of their psychosocial stress. In addition, timely, transdisciplinary (medical-psycho-social) attention to all relatives might prevent them from developing psychopathology like PTSD.

## Data Availability Statement

The raw data supporting the conclusions of this article will be available from the corresponding author on reasonable request.

## Ethics Statement

The studies involving human participants were reviewed and approved by the Vincent van Gogh Institutional Review Board (Decision letter references: JE/hr/2020.012/; JE/hr/2021.004). The participants provided their written informed consent to participate in this study.

## Author Contributions

AB-R, EW, LG, and JE designed and planned the study. AB-R and AB performed data collection and executed statistical analyses. AB-R drafted the manuscript. EW, LG, and JE revised and critically reviewed the manuscript for important content. All authors read and authorized the final version of the manuscript.

## Conflict of Interest

The authors declare that the research was conducted in the absence of any commercial or financial relationships that could be construed as a potential conflict of interest.

## Publisher's Note

All claims expressed in this article are solely those of the authors and do not necessarily represent those of their affiliated organizations, or those of the publisher, the editors and the reviewers. Any product that may be evaluated in this article, or claim that may be made by its manufacturer, is not guaranteed or endorsed by the publisher.
