# Supplementary material for: Family Matters: Trauma and Quality of Life in Family Members of Individuals With Prader-Willi Syndrome
Source: Front Psychiatry. 2022 Jun 28;13:897138. doi: 10.3389/fpsyt.2022.897138 (PMC9273751; doi:10.3389/fpsyt.2022.897138)
Supplement: Supplementary file 1 [file Table_1.docx]

**Supplementary List**

*Measurement Instrument PCL-5: Clusters and Items*

| Cluster B |  |  |
| --- | --- | --- |
|  | Item |  |
|  | 1 | Repeated, disturbing, and unwanted memories of the stressful experience |
|  | 2 | Repeated, disturbing dreams of the stressful experience |
|  | 3 | Suddenly feeling or acting as if the stressful experience were actually happening again (as if you were actually back there reliving it) |
|  | 4 | Feeling very upset when something reminded you of the stressful experience |
|  | 5 | Having strong physical reactions when something reminded you of the stressful experience (for example, heart pounding, trouble breathing, sweating) |
| Cluster C |  |  |
|  | 6 | Avoiding memories, thoughts, or feelings related to the stressful experience |
|  | 7 | Avoiding external reminders of the stressful experience (for example, people, places, conversations, activities, objects, or situations) |
| Cluster D |  |  |
|  | 8 | Trouble remembering important parts of the stressful experience |
|  | 9 | Having strong negative beliefs about yourself, other people, or the world (for example, having thoughts such as: I am bad, there is something seriously wrong with me, no one can be trusted, the world is completely dangerous) |
|  | 10 | Blaming yourself or someone else for the stressful experience or what happened after it |
|  | 11 | Having strong negative feelings such as fear, horror, anger, guilt, or shame |
|  | 12 | Loss of interest in activities that you used to enjoy |
|  | 13 | Feeling distant or cut off from other people |
|  | 14 | Trouble experiencing positive feelings (for example, being unable to feel happiness or have loving feelings for people close to you) |
| Cluster E |  |  |
|  | 15 | Irritable behavior, angry outbursts, or acting aggressively |
|  | 16 | Taking too many risks or doing things that could cause you harm |
|  | 17 | Being “superalert” or watchful or on guard |
|  | 18 | Feeling jumpy or easily startled |
|  | 19 | Having difficulty concentrating |
|  | 20 | Trouble falling or staying asleep |

*Note.* Derived from the PTSD Checklist for DSM-5 (PCL-5) with Life Events Checklist for DSM-5 (LEC-5) and Criterion A (Weathers et al., 2013). Version date: 11 April 2018

Available from <https://www.ptsd.va.gov/>
